# *Castanea crenata* honey reduces influenza infection by activating the innate immune response

**DOI:** 10.3389/fimmu.2023.1157506

**Published:** 2023-08-22

**Authors:** Eun-Bin Kwon, Se-Gun Kim, Young Soo Kim, Buyun Kim, Sang Mi Han, Hye Jin Lee, Hong Min Choi, Jang-Gi Choi

**Affiliations:** ^1^ Korean Medicine Application Center, Korea Institute of Oriental Medicine, Daegu, Republic of Korea; ^2^ Department of Agricultural Biology, National Institute of Agricultural Sciences, Rural Development Administration, Wanju, Republic of Korea

**Keywords:** influenza A virus, *Castanea crenata* honey, interferon, mitochondrial antiviral signaling protein, retinoic acid-inducible gene-1, IFN-inducible transmembrane 3

## Abstract

Influenza is an acute respiratory disorder caused by the influenza virus and is associated with prolonged hospitalization and high mortality rates in older individuals and chronically ill patients. Vaccination is the most effective preventive strategy for ameliorating seasonal influenza. However, the vaccine is not fully effective in cases of antigenic mismatch with the viral strains circulating in the community. The emergence of resistance to antiviral drugs aggravates the situation. Therefore, developing new vaccines and antiviral drugs is essential. *Castanea crenata* honey (CH) is an extensively cultivated food worldwide and has been used as a nutritional supplement or herbal medicine. However, the potential anti-influenza properties of CH remain unexplored. In this study, the *in vitro* and *in vivo* antiviral effects of CH were assessed. CH significantly prevented influenza virus infection in mouse Raw264.7 macrophages. CH pretreatment inhibited the expression of the viral proteins M2, PA, and PB1 and enhanced the secretion of proinflammatory cytokines and type-I interferon (IFN)-related proteins *in vitro*. CH increased the expression of RIG-1, mitochondrial antiviral signaling (MAVS) protein, and IFN-inducible transmembrane protein, which interferes with virus replication. CH reduced body weight loss by 20.9%, increased survival by 60%, and decreased viral replication and inflammatory response in the lungs of influenza A virus-infected mice. Therefore, CH stimulates an antiviral response in murine macrophages and mice by preventing viral infection through the RIG-1-mediated MAVS pathway. Further investigation is warranted to understand the molecular mechanisms involved in the protective effects of CH on influenza virus infection.

## 1 Introduction

The influenza virus causes a disease, infecting 1 billion people worldwide and resulting in 650,000 deaths annually. According to a previous report, influenza viruses frequently mutate and have a high potential to cause pandemics. Vaccines and the inhibitor of the influenza virus surface protein NA, Tamiflu^®^, are used for the prevention and treatment of influenza, respectively (1–3). However, resistance to drugs and the emergence of novel strains can reduce the effectiveness of prevention and treatment; therefore, new therapies are needed.

Innate immunity, the first line of host defense, efficiently and rapidly limits viral infection. An immune response in which foreign substances are destroyed directly without exposure to a specific antigen is known as innate or nonspecific immunity. Leukocytes involved in innate immunity include phagocytes such as neutrophils and macrophages (4, 5). Macrophages recognize nucleic acids (DNA and RNA) in viruses using pattern recognition receptors and secrete cytokines and interferons as antiviral agents. Representative pattern recognition receptors include toll-like receptor, C-type lectin receptor, retinoic acid-inducible gene-I-like receptor (RLR), nucleotide oligomerization domain-like receptor, and absent in melanoma-like receptor. Members of the RLR family, including RIG-1, melanoma differentiation factor 5, and laboratory of genetics and physiology 2, can detect viral RNA ligands or processed self-RNA in the cytosol to induce innate immunity, inflammation, and gene expression, thereby limiting the infection. Activated RLR transduces the signal to the mitochondrial antiviral signaling (MAVS) protein, which activates intranuclear translocation of interferon regulatory transcription factor (IRF) 3 and 7. NF-κB induces type-1 interferon (IFN) and proinflammatory cytokine production. IFNs limit viral infection and enhance cell-mediated immunity against intracellular strains (6–10). IFN molecules bind to cell surface receptors and initiate a signaling cascade through the Janus kinase signal transducer and activator of transcription pathway, leading to the transcriptional regulation of hundreds of IFN-regulated genes that comprise IFN-stimulated genes (ISGs). ISGs, members of the IFN-inducible transmembrane (IFITM) family, block the viral entry. Antiviral signaling proteins stimulated by IFNs bind to viral mRNA to inhibit transcription or cause viral protein degradation, ultimately suppressing virus proliferation and promoting virus death. Type-1 IFN production through the RLR signaling pathway plays an important role in antiviral function (11). Therefore, increasing innate immunity by inducing IFN production is an attractive target in antiviral research.

Based on their stability and efficacy, traditional natural products have been identified to play a vital role in drug development for various diseases. Studies have identified antiviral substances derived from natural products that enhance innate immunity. Recently, it was demonstrated that *Panax notoginseng* extract and *Tilia amurensis* honey inhibit influenza A virus (IAV) infection by increasing IFN production in Raw264.7 macrophages. (12–14) Efforts have been made to identify new materials using food ingredients with established safety profiles, thus developing effective and safe antiviral treatments for IAV.

Honey is derived from the flower nectar of various plant species. It has been used extensively in traditional medicine for treating bronchial asthma, sore throats, tuberculosis, fatigue, dizziness, hepatitis, constipation, and wounds. Recent studies have demonstrated its efficacy in managing conditions such as diabetes, cancer, asthma, and cardiovascular diseases. These studies considered the most popular varieties, manuka and kanuka honey (15, 16). However, there is a lack of scientific research on the efficacy of *Castanea crenata* honey (CH). CH is predominantly obtained from the flower nectar of the Castanea crenata tree via bees. CH exhibits a dark brown color, mild sweetness, complex flavor, intense aroma, and slightly pungent, spicy persistent taste. CH boasts a diverse nutritional profile, containing higher quantities of vitamins and minerals than other honey varieties (17). Notably, it is a source of vitamin C, potassium, manganese, iron, copper, trace amounts of amino acids, and other essential nutrients.

However, there is a lack of research on the specific mechanism by which CH exerts its antiviral effects through immune enhancement. Therefore, we aimed to determine whether CH inhibits influenza infection by increasing IFN production in mouse macrophages through RLR-mediated MAVS activation, an antiviral mechanism associated with innate immunity.

## 2 Materials and methods

### 2.1 Reagents

Dimethyl sulfoxide, and human and mouse IFN-β were purchased from Sigma-Aldrich (Saint Louis, MO, USA). Chicken red blood cells were obtained from Innovative Research Inc. (Southfield, MI, USA). Pro-Prep protein extraction solution was purchased from Intron Biotechnology (Seoul, Korea). NP, NA, HA, M2, and PB1 antibodies were obtained from GeneTex (Irvine, CA, USA). CH was purchased from the Korea Apicultural Agriculture Cooperative (Anseong, Korea) in 2020. The honey sample was stored at room temperature under dark conditions until the experiments.

### 2.2 Cells and viruses

Raw264.7 cells (murine macrophages) were purchased from the American Type Culture Collection (Manassas, VA, USA). The cells were cultured in DMEM supplemented with 10% fetal bovine serum and 1% antibiotic–antimycotic at 37°C under 5% CO2. A green fluorescent protein (GFP)-encoding influenza A strain, A/PR8/34-GFP, and Puerto Rico/8/34 virus were used as described in previous studies (18).

### 2.3 Antiviral assay

Raw264.7 cells were cultured in 24-well plates at a density of 1 × 10^5^ cells/well for 18 h. The cells were pretreated with CH for 18 h and then infected with IAV (MOI = 2) for 24 h. Mouse IFN-β was used as a positive control. After the infection, viral GFP expression was estimated using fluorescence microscopy (Nikon, Tokyo, Japan), and quantitative analysis was performed via flow cytometry (CytoFLEX; Beckman Coulter Inc., Pasadena, CA, USA).

### 2.4 RNA interference

The cells were cultured for 18h until 60% confluency was achieved. MAVS and negative control siRNA were purchased from Bioneer (Deageon, Korea). Transient transfections were performed using the Lipofectamine RNAiMAX reagent according to the manufacturer’s instructions. Briefly, 5 μL of siRNA and 5 μL of Lipofectamine RNAiMAX were mixed with 90 μL of Opti-MEM. The mixture was incubated at room temperature for 20 min. After the reaction, the mixture was added dropwise to culture wells containing Opti-MEM. After 4 h, the medium was replenished with fresh DMEM.

### 2.5 Real-time reverse transcription polymerase chain reaction

Total RNA was extracted using TRIzol kit according to the manufacturer’s instructions. cDNA synthesis was performed using Easy-BLUE RNA extraction kit (iNtRON Biotech), AccuPower CycleScript RT PreMix (Bioneer, Daejeon, South Korea), and CFX96 Touch Real-Time polymerase chain reaction system (Bio-Rad, Hercules, CA, USA) according to the manufacturer’s instructions.

### 2.6 Western blotting

Cell lysates were prepared using the PRO-PREP protein extraction solution. Protein was quantified using the Bradford method. Proteins were separated using 8%–15% sodium dodecyl sulfate–polyacrylamide gel electrophoresis, after which they were transferred to polyvinylidene fluoride membranes. The membranes were blocked with 0.5× Ez-Block Chemi (Amherst, MA, USA) and incubated with antibodies. Signals were detected using the ChemiDoc imaging system (UVITEC, Cleaver Scientific Ltd, UK) equipped with an enhanced chemiluminescence reagent (Thermo Scientific, Rockford, IL, USA). Relative protein band intensities were determined using ImageJ software.

### 2.7 Immunofluorescence

Immunofluorescence (IF) was performed as follows. Coverslips coated with poly-D-lysine (Gibco, USA) were incubated for 30 min at 37°C. Raw264.7 cells were seeded at a density of 1 × 10^5^ cells per slide and incubated for 24 h. The cells were treated with CH for 24 h, fixed at room temperature with 4% paraformaldehyde in phosphate-buffered saline (PBS) for 30 min, and washed with PBS 4 times. The cells were incubated for 30 min with a blocking solution (0.5% Ez-Block Chemi) and incubated overnight at 4°C for 24 h with antibodies against PB1, p-IRF3, and MAVS. The cells were washed with PBS, incubated for 30 min with secondary antibody and lysotracker, washed with PBS, and stained with Hoechst 33342 for 5 min. The coverslips were mounted onto slides with mounting medium and visualized via fluorescence microscopy (Lionheart FX automated microscopy, BioTek, Vermont, USA). Quantitative analysis was performed using flow cytometry.

### 2.8 Electrophoretic mobility shift assay (EMSA)

Nuclear extracts were prepared as described previously (19). EMSA was performed to determine the DNA-binding activity of NF-κB in influenza-infected cells with a DIG-labeled oligonucleotide (NF-κB: 5′-AGT TGA GGG GAC TTT CCC AGG C-3′) using nonradioactive EMSA kit (Roche, Mannheim, Germany). The nuclear extracts and labeled oligonucleotide (Invitrogen) were incubated at room temperature for 1 h in binding buffer. The mixture was electrophoresed on a 6% nondenatured polyacrylamide gel and then electrotransferred to a positively charged nylon membrane. Subsequently, the membrane was treated with anti-digoxigenin-Ap Fab fragments (Roche), and the chemiluminescent substrate CSPD (Roche) was added. Chemiluminescence was detected via autoradiography. The specificity of the NF-κB-binding complex was determined via the addition of excess unlabeled oligonucleotides.

### 2.9 Animals

This study followed the guidelines of and was approved by the Institutional Animal Care and Use Committee of the Laboratory Animal Center of Daegu-Gyeongbuk Medical Innovation Foundation (DGMIF- 22030103-01). Female 5-week-old BALB/c mice obtained from Orient Bio Inc. (Seongnam, South Korea) were acclimated for at least 1 week under standard housing conditions. Standard rodent chow and water were provided ad libitum. The mice were divided into 4 groups, with 10 mice in each group. The group without viral infection was used as a negative control. The mice in the experimental groups were orally administered with PBS or 300 or 600 mg/kg CH at a total volume of 200 μL once daily for 14 days before inducing IAV infection. The mice were infected intranasally with 20 µL of A/PR/8/34 in PBS at 50% lethal mouse dose (LD50). Then, survival rates and body weights were measured. Survival was monitored for 10 days post-infection (dpi) at fixed time points. At 6 dpi, three mice were randomly selected from each group and euthanized to perform lung histopathology. The lung tissues were immediately fixed with 10% formalin in paraffin-embedded neutral buffer and sliced into 4–6-µm sections using a microtome. The sections were mounted on a slide, stained with eosin, and examined via optical microscopy. Survival in the remaining mice was measured at 10 dpi.

The immune response animal experiments (reference number #D-21–069) followed the guidelines of and were approved by the Animal Care and Use Committee of the Korea Institute of Oriental Medicine (KIOM, Daejeon, Korea). Female 5-week-old BALB/c mice from Orient Bio Inc. (Seongnam, South Korea) were acclimated for at least 1 week under standard housing conditions at KIOM. The mice were categorized into three experimental groups. The mice in the experimental group were orally administered with PBS or 300 or 600 mg/kg CH at a total volume of 200 μL once daily for 14 days. At 14 dpi, six mice from each group were randomly selected and euthanized to evaluate cytokines and isolated splenocytes. Splenocytes (1 × 105 cells/well) were added into 24-well plates containing YAC-1 cells (1 × 10^4^ cells/well); then, the amount of lactate dehydrogenase liberated from the target cells due to spleen natural killer cell activity was measured using the lactate dehydrogenase Cytotoxicity Assay Kit II (Abcam, Cambridge, UK) according to the manufacturer’s instructions.

### 2.10 Statistical analysis

Data are presented as the mean ± standard error of mean. Differences between the mean values of treated and control groups were analyzed for significance using one-way analysis of variance. For multigroup comparisons, Tukey’s *post hoc* test was performed. All analyses were performed using GraphPad PRISM v.5.02 (GraphPad, USA). P-values of <0.05 were considered to indicate statistical significance.

## 3 Results

### 3.1 Antiviral effect of CH in Raw264.7 cells

**Figure 1:**
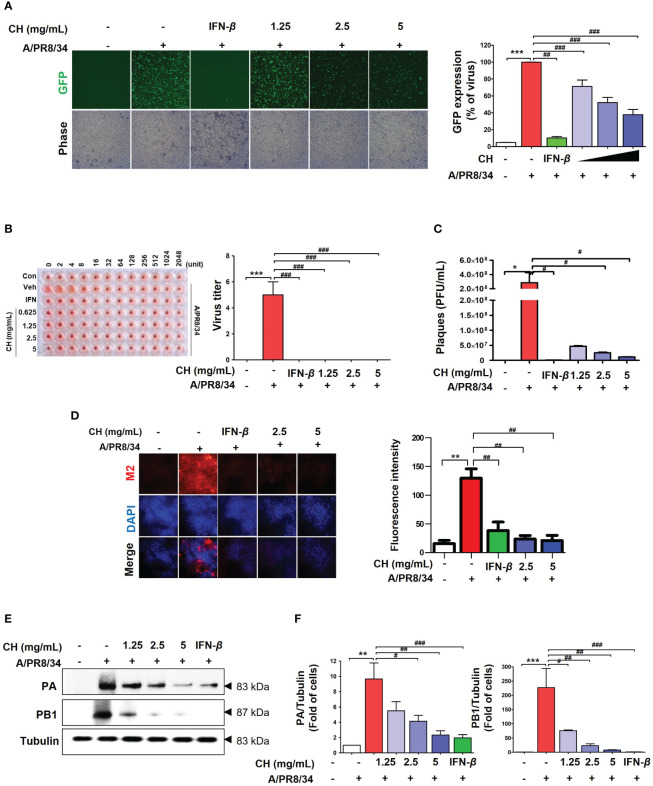
Antiviral effect of CH on IAV-infected Raw264.7 cells. The cells were pretreated with CH (1.25, 2.5, and 5 mg/mL) or 1,000 units of IFN-β as a positive control for 24 h before infection with IAV-GFP. Detection of GFP using **(A)** fluorescence microscopy (10×) and flow cytometry. **(B)** Infected supernatant was added to round plates and then diluted with PBS. Finally, 0.5% cRBC in PBS was added to each well and incubated for 1 h at 25°C before imaging. **(C)** MDCK cells were infected with supernatant including virus and concentration of CH for 2h at 37°C. After incubation, cell monolayer was covered with 1.5% agarose and 2X DMEM containing 10% FBS and 1% P/S for 36h. And, cell monolayer was fixed with 10% formalin, stained with 1% crystal violet, and plaques were counted. **(D)** M2 viral protein expression was measured using immunofluorescence. **(E)** Western blot analysis of PA and PB1 expression and **(F)** quantitative analysis of the protein bands using ImageJ software. Three independent experiments were performed. Bar graph (mean ± standard error of mean) statistical data were determined using one-way analysis of variance with Tukey’s *post hoc* test. ^***^P < 0.001 and ^**^P < 0.01 compared with the untreated group (Con). ^###^P < 0.001; ^##^P < 0.01; ^#^P < 0.05, compared with the virus-infected group (Veh). CH, chestnut honey; IAV, influenza A virus; IFN, interferon; CH stimulates the production of cytokines in Raw264.7 cells.

**Figure 2:**
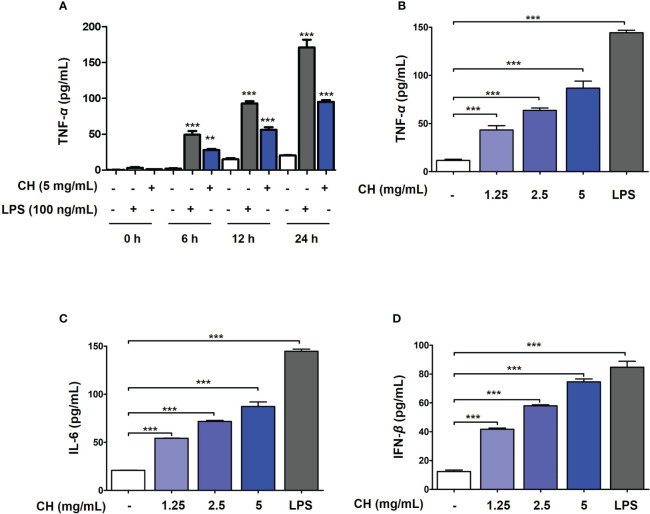
CH induces the production of proinflammatory cytokines and IFN-β in Raw264.7 cells. **(A)** The cells were treated with CH or LPS at the time (0, 6, 12 and 24 hours). After incubation, the media was harvest and measured by secretion of TNF-α using ELISA kit. The cells were pretreated with CH (1.25, 2.5, and 5 mg/mL) or 100 ng/mL LPS as a positive control for 24 h. The supernatants were collected and centrifuged at 1500 rpm for 5 min at 4°C. To measure cytokine secretion, supernatants were distributed into **(B)** tumor necrosis factor (TNF)-α, **(C)** murine interleukin (IL)-6, and **(D)** IFN-β capture antibody-coated enzyme-linked immunosorbent assay plates. Three independent experiments were performed. Bar graph (mean ± standard error of mean) statistical data were determined using one-way analysis of variance with Tukey’s *post hoc* test. ^***^P < 0.001 and ^**^P < 0.01 compared with the untreated group (Con).

We investigated whether CH can inhibit IAV infection *in vitro*. Before testing the antiviral activity of CH, we evaluated its toxicity. CH exhibited no cytotoxicity at concentrations of 1.25, 2.5, and 5 mg/mL in Raw 264.7 cells (**Supplementary Figure 1**). The antiviral activity of CH was investigated based on the suppression of A/PR/8/34-GFP infection in Raw264.7 cells through GFP measurement. Compared with the virus-infected group (Veh), CH pretreatment at 1.25, 2.5, and 5 mg/mL decreased A/PR/8/34-GFP infection in Raw264.7 cells by 16.9%, 38.3%, and 53%, respectively (**Figure 1A**). Additionally, the hemagglutinin (HA) assay was used to quantify viral titers in the supernatant. CH significantly decreased hemagglutination (**Figure 1B**). In addition, we evaluate virus titer using by plaque assay. As show **Figure 2D**, CH significantly reduced plaque production (**Figure 1C**). IF and western blotting were performed to evaluate the expression of the viral proteins M2, PB1, and PA, which was shown to be suppressed by CH (**Figures 1D–F**). These results indicate that CH pretreatment significantly inhibits IAV infection (**Supplementary Figure 2**) and viral protein expression in Raw264.7 cells.

### 3.2 CH reduced inflammation response by infection of IAV in Raw264.7 cells

**Figure 3:**
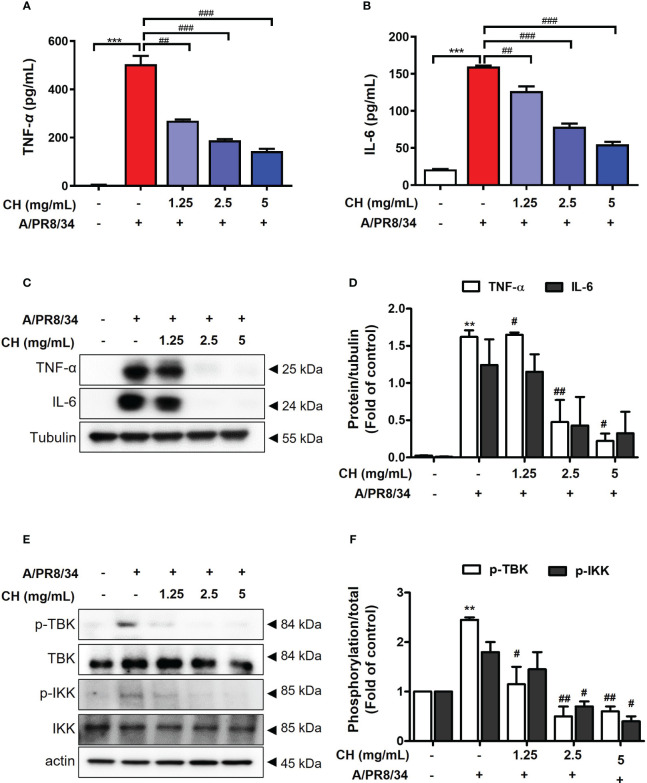
Effect of CH on virus induced inflammation. The cells were pretreated with CH for 24 h. After incubation, cells were harvested 24 h after IAV infection. The supernatants were collected and centrifuged at 1500 rpm for 5 min at 4°C. Supernatants were distributed into **(A)** tumor necrosis factor (TNF)-α and **(B)** murine interleukin (IL)-6 capture antibody-coated enzyme-linked immunosorbent assay plates to measure cytokine secretion. **(C)** Western blot analysis of TNF-α and IL-6 using whole-cell lysates and **(D)** quantitative analysis of protein bands using the ImageJ software. **(E)** Expression of protein including phosphorylation TBK and IKK were analyzed by western blot. **(F)** Quantitative analysis of protein bands using the ImageJ software Bar graph (mean ± SEM) statistics were determined using one-way ANOVA with Tukey’s *post hoc* test. ^***^P < 0.001 and **P < 0.01 compared with the untreated group (Con). ^###^P < 0.001; ^##^P < 0.01; ^#^P < 0.05 compared with the virus-infected group (Veh). CH, Chestnut honey; IAV, Influenza A virus; IFN, interferon.

It was confirmed that influenza A virus infection was inhibited by CH pretreatment. Viral infection within the host cell increases the inflammatory response, causing various damages such as intracellular hyperinflammatory response and cell death (20, 21). Therefore, to evaluate the effect of CH on the increased inflammatory response after viral infection, Raw264.7 cells were pretreated with CH at different concentrations and then infected with the virus. After the reaction, TNF-α and IL-6 were measured in the supernatant using the ELISA method, and the proteins were isolated to confirm the expression of phosphorylated TBK and IKK in the cells. As shown in **Figures 3A–D**, CH was shown to reduce the secretion and intracellular expression of TNF-α and IL-6, which are inflammatory cytokines that increase after viral infection. In addition, the phosphorylation levels of TBK and IKK among signal transduction pathways related to the inflammatory response were investigated. The results shown that phosphorylation of TBK and IKK, which had been increased by viral infection, was decreased by CH treatment (**Figures 3E, F**). The data suggest that CH treatment inhibits signaling pathways that increase secretion of inflammatory cytokines. Taken together, this research was indicating that CH alleviates the inflammatory response increased by the virus.

### 3.3 CH stimulates the production of cytokines in Raw264.7 cells

To determine the mechanism by which CH inhibits viral infection, we first investigated innate immune factors in Raw264.7 cells. Proinflammatory cytokines, including tumor necrosis factor (TNF)-α, interleukin (IL)-6, and type-1 IFN, are essential for inducing immunoregulatory activity and antiviral responses (2). To determine whether the antiviral activity of CH is due to the increase in innate immunity through the production of proinflammatory cytokines and type-1 IFN, we evaluated the levels of TNF-α, IL-6, and type-1 IFN using ELISA. Prior to the experiment, endotoxin contained in CH was measured. Since endotoxin affects innate immune factors, the experiment was conducted after examining the endotoxin content of CH. In general, all honey solutions used in this study had endotoxin levels of < 0.5 ng/mL (20, 22). After confirming that CH have amount of endotoxin at 0.2 ng/mL (**Table S1** and **Figure S3**), and then the amount of innate immune factor secretion was measured. Frist, secretion of pro-inflammatory cytokines such as TNF-α was analyzed based on the duration of CH treatment (0, 6, 12 and 24 hours) in Raw264.7 cells. As shown in **Figure 2A**, it was confirmed that CH and LPS increased the secretion of TNF-alpha over time. After, we determined that secretion of innate immune factor (TNF-α, IL-6 and IFN-β) according to the concentration of CH used. The results shown that compared with the control group, TNF-α and IL-6 levels increased dose-dependently in the CH-treated group (**Figures 2B, C**). Furthermore, CH treatment stimulated the secretion of IFN-β (**Figure 2D**). These results indicate that CH induces an antiviral response mediated by proinflammatory cytokines and IFN-β in murine macrophages.

### 3.4 CH activates IRF-3 and NF-κB signaling

**Figure 4:**
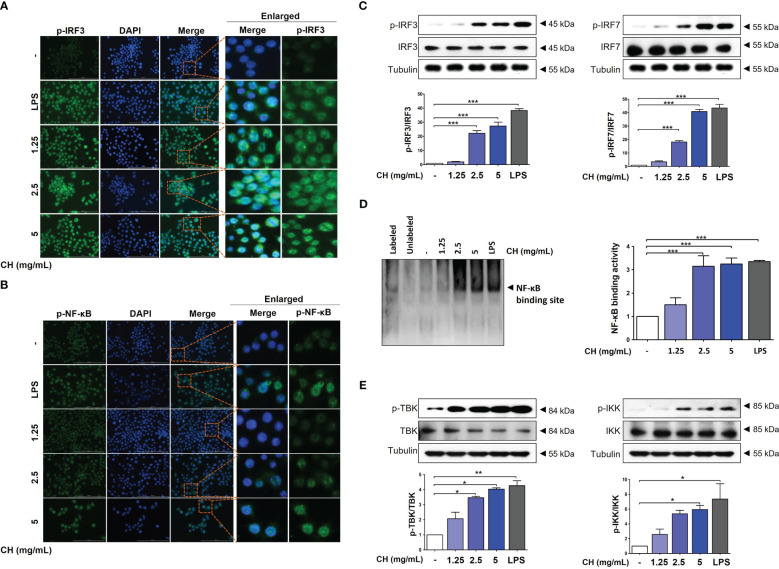
CH increases the phosphorylation of IRF-3 and NF-κB. The cells were pretreated with CH (1.25, 2.5, and 5 mg/mL) or 100 ng/mL LPS as a positive control for 24 h. Expression of **(A)** p-IRF3 and **(B)** p-NF-κB was detected via immunofluorescence. **(C)** Western blot analysis of IRF3 and IRF7 using whole-cell lysates and quantitative analysis of protein bands were performed via ImageJ software. **(D)** The DNA-binding activity of NF-κB in nuclear extracts was measured using EMSA. **(E)** Western blot analysis of TBK and IKK using whole-cell lysates and quantitative analysis of protein bands were performed via ImageJ software. Three independent experiments were performed. Bar graph (mean ± standard error of mean) statistical data were determined using one-way analysis of variance with Tukey’s *post hoc* test^. ***^P < 0.001, ^**^P < 0.01, and ^*^P < 0.05 compared with the untreated group (Con).

IRF3 and NF-κB are transcriptional regulators of cellular responses in various cell types important for innate immune responses. Studies have reported that IRF3 and NF-κB are required for protecting host cells against viral infection. Phosphorylation of IRF3 and NF-κB leads to their translocation into the nucleus, activating the production of IFNs (23, 24). Analysis using immunofluorescence (IF) shown that CH treatment promoted expression of p-IRF3 and p-NF-κB in nuclear, as depicted in **Figures 4A, B**, respectively. We can speculate that the enhanced expression of p-IRF3 and p-NF-kB in the nucleus leads to an increased translocation of IRF3 and NF-kB. However, it is necessary to verify whether the expression in the cytoplasm is indeed reduced accurately. Moreover, cells pretreated with CH showed increased phosphorylation of IRF3 and IRF7 (**Figure 4C**). We analyzed the nuclear extracts of Raw264.7 cells incubated with CH using DIG-labeled oligonucleotides corresponding to NF-κB-binding sites. The formation of the NF-κB–DNA complex was prominent in nuclear extracts obtained from CH-treated cells (**Figure 4D**) (25). Additionally, we analyzed the phosphorylation of TBK and IKK, which upregulate the expression of IRF3 and NF-κB. CH treatment increased the phosphorylation of IKK and TBK in Raw264.7 cells (**Figure 4E**). These results suggest that CH induces type-1 IFN gene transcription to elicit an antiviral response.

### 3.5 CH activates the RIG-MAVS complex in Raw264.7 cells

**Figure 5:**
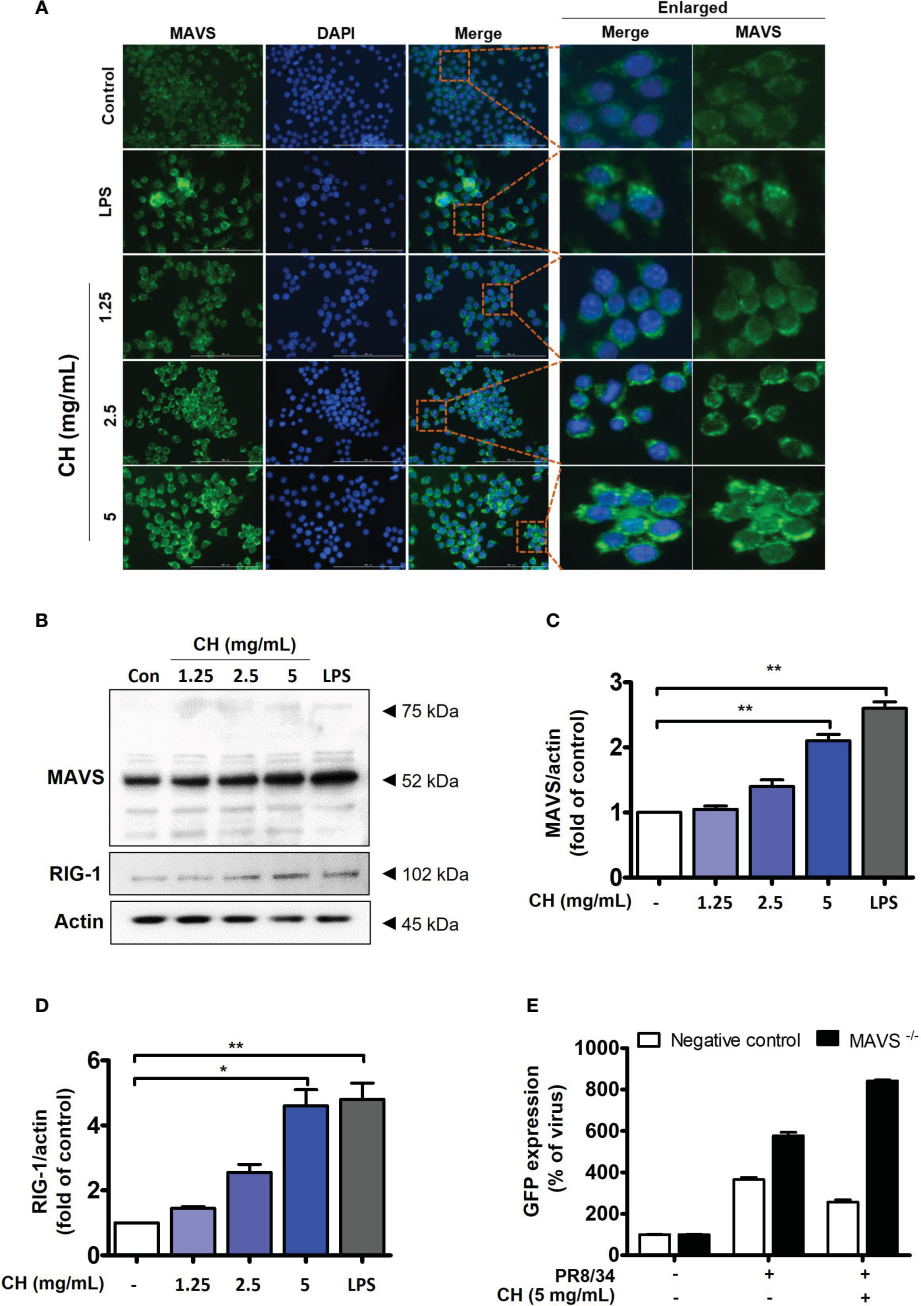
CH activates the RIG-1-mediated MAVS pathway. The cells were pretreated with CH (1.25, 2.5, and 5 mg/mL) or 100 ng/mL LPS as a positive control for 24 h. **(A)** Expression of MAVS was detected via immunofluorescence. **(B)** Western blot analysis of RIG-1 and MAVS using whole-cell lysates and **(C, D)** quantitative analysis of protein bands (MAVS and RIG-1) was performed via ImageJ software. **(E)** Cells were transfected with siRNA against MAVS and then infected with GFP-IAV, followed by detection of viral GFP via flow cytometry. Bar graph (mean ± standard error of mean) statistical data were determined using one-way analysis of variance with Tukey’s post hoc test. **P < 0.01 and *P < 0.05 compared with the untreated group (Con).

Upon viral recognition, RIG-1 interacts with MAVS, which induces the phosphorylation of IKK and TBK, leading to the activation of the transcription factors IRF3 and NF-κB (23). We previously confirmed that CH increases the phosphorylation of IKK and TBK (**Figure 4**). Therefore, we investigated the expression of RIG and MAVS, which are located upstream of IKK and TBK. IF and western blotting were performed to assess the expression of MAVS. As shown in **Figures 5A–D**, CH increased the expression of MAVS in Raw264.7 cells. Whereas, MAVS, RIG and related signals showed decreased expression after virus infection. The data suggest that protein expression was reduced because treatment of CH reduced viral infection (**Figure S4**). Furthermore, we knocked down MAVS using siRNA to provide evidence that CH activates the MAVS signaling pathway. When cells with suppressed MAVS expression were infected with the influenza virus, the MAVS knockdown group exhibited higher susceptibility to influenza virus infection than the negative control group. Moreover, when the cells were infected with the influenza virus after treatment with CH, the antiviral effect of CH was not observed in the MAVS knockdown group. The IAV infection inhibitory effect of CH significantly increased after MAVS knockdown (**Figure 5E**).

### 3.6 CH increases antiviral signaling pathways in Raw264.7 cells

**Figure 6:**
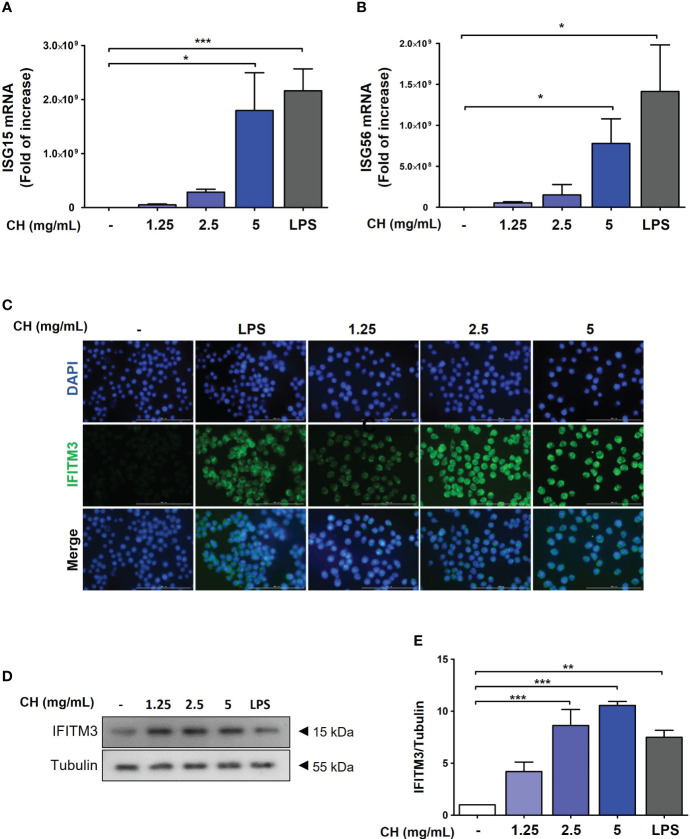
CH stimulates the expression of antiviral genes. The cells were pretreated with CH (1.25, 2.5, and 5 mg/mL) or 100 ng/mL LPS as a positive control for 24 h. RNA was isolated using TRIzol kit, and the expression of **(A)** ISG15 and **(B)** ISG56 was detected. **(C)** Expression of IFITM3 was detected via immunofluorescence. **(D)** Western blot analysis of IFITM3 using whole-cell lysates and **(E)** quantitative analysis of protein bands were performed via ImageJ software. Bar graph (mean ± standard error of mean) statistical data were determined using one-way analysis of variance with Tukey’s *post hoc* test. ^***^P < 0.001, ^**^P < 0.01, and ^*^P < 0.05 compared with the untreated group (Con).

Type-1 IFN induces the activation of ISG transcription factors, resulting in the induction of ISG15 and ISG56, which are associated with the increased expression of IFITMs (26, 27). To assess whether CH enhanced the expression of ISG15 and ISG56, we performed reverse transcription polymerase chain reaction. The results demonstrated that compared with the control group, CH significantly increased the expression of ISG15 and ISG56 in a dose-dependent manner (**Figures 6A, B**). Furthermore, IF and western blot results revealed that CH stimulates the expression of IFITM3 in a dose-dependent manner (**Figures 6C–E**). Taken together, these results indicate that the expression of antiviral genes that were induced by CH can effectively hinder viral entry and replication.

### 3.7 Protective effect of CH on IAV in BALB/c mice

**Figure 7:**
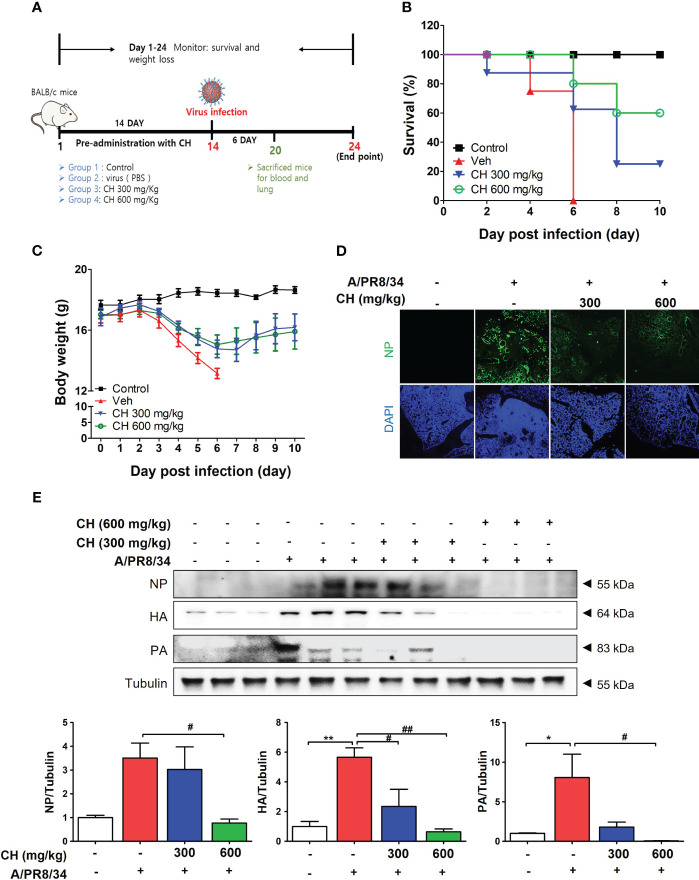
Antiviral effect of CH on IAV-infected BALB/c mice. **(A)** Schematic of the animal experiments. IAV was administered into the mouse nasal cavities after oral administration with 300 or 600 mg/kg CH. **(B)** Survival rate and **(C)** body weight was monitored daily until 10 dpi. **(D)** Immunofluorescence analysis revealed the levels of IAV NP in virus-infected mouse lungs. Scale bar = 100 mm. **(E)** Western blot analysis of NP, HA, and PA expression and quantitative analysis of the protein bands were performed using ImageJ software. Bar graph (mean ± standard error of mean) statistical data were determined using one-way analysis of variance with Tukey’s *post hoc* test. ^**^P < 0.01 and *P < 0.05 compared with the untreated group (Con). ^##^P < 0.01 and ^#^P < 0.05, compared with the virus-infected group (Veh).

In this study, we investigated the antiviral effects of CH against IAV infection in BALB/c mice. The mice were orally administered with CH daily for 2 weeks and then infected with IAV (**Figure 7A**). We monitored their survival rates and body weights throughout the experiment. Mice pretreated with CH or PBS did not show significant differences in body weight (**Figure S5**). However, IAV-infected mice experienced significant body weight loss and succumbed to death within 6 day post infection (dpi) (**Figure 7B**). In contrast, CH-treated mice exhibited higher survival rates in a dose-dependent manner and maintained higher body weights than IAV-infected mice (**Figure 7C**). We evaluated the expression of viral proteins in dissected mouse lungs (n = 3) using IF and western blotting. NP, PB1, HA, and NA levels decreased in the lungs of CH-treated mice compared with those in IAV-infected mice (**Figures 7D, E**).

**Figure 8:**
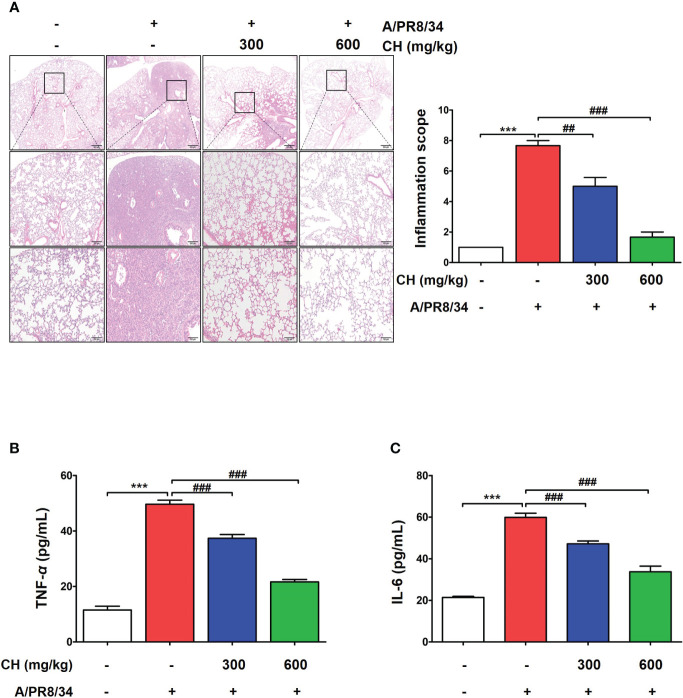
Effects of CH on inflammation in IAV-infected BALB/c mice. **(A)** A representative hematoxylin and eosin image showing histopathological damage in sectioned lung tissue of untreated and CH-treated mice. **(B)** Serum TNF-α and **(C)** IL-6 levels were measured using ELISA. The experiment was performed three times. Bar graph (mean ± standard error of mean) statistical data were determined using one-way analysis of variance with Tukey’s *post hoc* test. ^***^P < 0.001 compared with the untreated group (Con). ^###^P < 0.001 and ^##^P < 0.01 compared with the virus-infected group (Veh).

**Figure 9:**
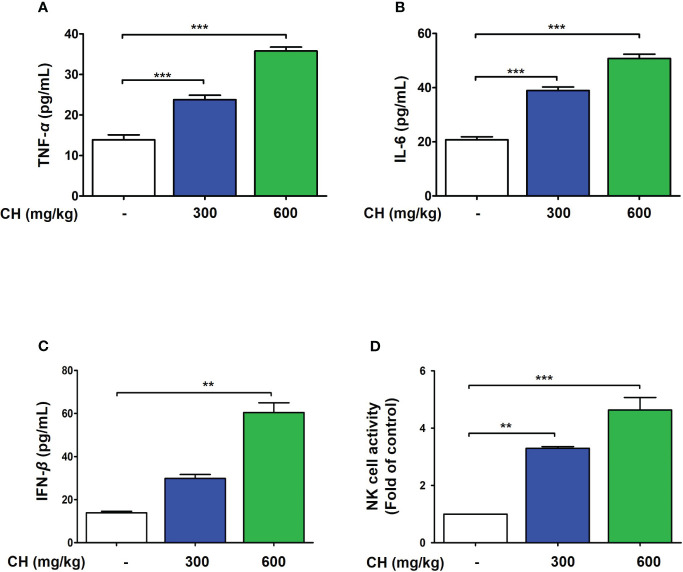
CH enhances the immune response in BALB/c mice. Mice were orally administered with 300 or 600 mg/kg CH for 2 weeks. **(A)** Serum TNF-α, **(B)** IL-6, and **(C)** IFN-β levels were measured using ELISA. **(D)** Splenocytes were incubated with YAC-1 cells, and the level of lactate dehydrogenase (LDH) released was determined using the LDH cytotoxicity assay. Bar graph (mean ± standard error of mean) statistical data were determined using one-way analysis of variance with Tukey’s *post hoc* test. ^***^P < 0.001 and ^**^P < 0.01 compared with the untreated group (Con).

These results suggest that CH confers protection against IAV infection in mice. Mouse lungs were sampled at 6 dpi and stained with hematoxylin and eosin to evaluate histopathological changes due to viral infection. Histopathological sections revealed marked thickening of bronchial epithelial cells and alveolar walls, resulting in pulmonary congestion in IAV-infected mice compared with controls. These changes were alleviated by CH administration (**Figure 8A**). Additionally, the increase in the serum levels of cytokines, such as TNF-α and IL-6, after IAV infection reduced significantly upon CH treatment (**Figures 8B, C**). These results indicate that CH alleviates the increase in lung inflammation due to viral infection. We isolated the spleen and blood of mice administered with CH for 14 days and evaluated the immune response. We revealed that the serum levels of TNF-α, IL-6, and IFN-β increased in a dose-dependent manner in mice administered with CH for 14 days (**Figures 9A–C**). Additionally, CH-administered mouse splenocytes showed significantly enhanced natural killer cell activity against YAC-1 cells at an effector cell–target cell ratio of 10:1 (**Figure 9D**).

Taken together, our findings demonstrate that CH administration confers protection against IAV infection in mice by promoting survival, maintaining body weight, reducing viral protein expression, alleviating lung inflammation and histopathological changes, and enhancing the immune response. These results support the potential use of CH as a natural antiviral agent for the prevention and treatment of IAV infection.

### 3.8 KYNA against IAV in Raw264.7 cells

**Figure 10:**
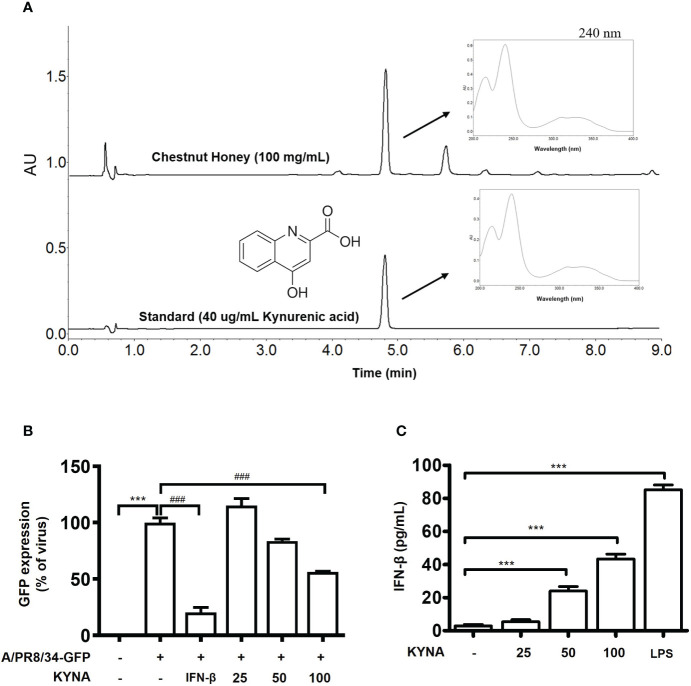
Antiviral effect of kynurenic acid (KYNA) component in CH. **(A)** UPLC **(B)** Antiviral effect of KYNA in CH in IAV-infected cells. The cells were pre-treated with concentration of KYNA before infection of IAV. 1,000 units of IFN-β as a positive control for 24 h before infection with IAV-GFP. Detection of GFP using flow cytometry. **(C)** The cells were treated with concentration of KYNA and 100 ng/L of LPS for 24 h. after incubation, secretion of IFN-beta was measured by ELISA using the supernatant. Bar graph (mean ± SEM) statistics were determined using one-way ANOVA with Tukey’s *post hoc* test. ^***^P < 0.001 compared with the untreated group (Con). ^###^P < 0.001 compared with the virus-infected group (Veh). CH, Chestnut honey; IAV, Influenza A virus; IFN, interferon.

There is a tendency to set kynurenic acid (KYNA), one of the quinoline alkaloids specifically contained mainly in chestnut honey, as an indicator substance for chestnut honey as an identify potential marker. Therefore, the kynurenic acid content of chestnut honey was analyzed according to the UPLC rapid analysis method of previously report (19). CH contains various components such as sugars and minerals, and KYNA was identified as a biologically active and marker compound of CH (**Figure 10A**). Therefore, we aimed to investigate whether the antiviral effect of chestnut honey was caused by KYNA. The results shown that KYNA have antiviral effect against IAV in dose-dependent manner (**Figure 10B**). In addition, it was determined whether KYNA promotes innate immune factors such as IFN-β. It was confirmed that KYNA increases the secretion of IFN-β in a dose-dependent manner (**Figure 10C**). Therefore, the results proved that KYNA, the main component of CH, shows antiviral effect by increasing innate immunity.

## Discussion

In this study, we revealed that CH exerts its antiviral effect by enhancing innate immunity. We treated Raw264.7 macrophages with CH and observed an increase in the secretion of proinflammatory cytokines and IFN-β. This suggests that CH stimulates the immune response and promotes the production of immune signaling molecules.

Honey exhibits immunomodulatory activities, indicating that it can influence and regulate immune responses (20, 28). These activities involve various mechanisms and honey components. New Zealand honey varieties, such as manuka and kanuka, have been extensively studied and demonstrated to possess anti-inflammatory and antibacterial activities(29). New Zealand honey varieties have been shown to reduce inflammation by inhibiting the production of proinflammatory cytokines such as TNF-α, IL-1β, and IL-6 and promoting the release of anti-inflammatory cytokines such as IL-10(28). This helps balance the immune response and alleviate excessive inflammation. IAV induces a robust inflammatory response following infection, which is important for the control of viral proliferation; it is associated with lung damage, morbidity, and death. Consequently, the effective management of inflammation after influenza virus infection is crucial. CH has been shown to reduce the levels of inflammatory factors that were induced by viral infection; moreover, it suppressed the expression of IRF3/7, TBK, and IKK (**Supplementary Figure 4**). These results suggest that CH alleviates the increased inflammatory response caused by viral infection by inhibiting viral infection. In this study, the anti-inflammatory effect of honey, particularly CH, was confirmed. By demonstrating the ability of CH to alleviate the inflammatory response, this study highlights the potential use of honey as an anti-inflammatory agent. This further supports the potential therapeutic applications of honey in managing inflammatory conditions that are associated with viral infection. Studies have demonstrated the ability of manuka and other honey varieties to induce proinflammatory cytokines, including TNF-α, IL-1β, and IL-6, via macrophages; stimulate natural killer cell activity; and regulate the proliferation and differentiation of immune cells.

Previous reports have shown that the immunomodulatory effects of honey are indistinguishable from those of endotoxins(30). Endotoxins are bacterial toxins released from the cell walls of certain bacteria, primarily gram-negative bacteria. These toxins, also known as lipopolysaccharides, can elicit inflammatory responses in the body and may affect the immune function. Previous studies have reported that natural honey contains substantial amounts of endotoxin, and the responses observed in the cell-based assays were similar to those induced by endotoxin alone. Therefore, we evaluated the endotoxin content of honey, including CH, oilseed rape honey, and acacia honey, using the Limulus amebocyte lysate assay. CH contained 0.2 ng/mL lipopolysaccharides and the remaining honey solutions contained < 0.1 ng/mL lipopolysaccharides (**Table S1**). According to (20, 22), young manuka and kanuka honey contained 0.03 and 0.5 ng/mL lipopolysaccharides, respectively. In general, all honey solutions used in this study had endotoxin levels of < 0.5 ng/mL. Although endotoxins may be present in honey, their levels are generally low, and the immunomodulatory properties of honey provide beneficial effects. Honey possesses inherent immunomodulatory properties that are not solely attributable to endotoxins. The diverse range of bioactive compounds present in honey contributes to its immunomodulatory effects, influencing immune responses through various mechanisms. Honey contains a complex mixture of bioactive compounds, including polyphenols, flavonoids, enzymes, peptides, and organic acids, which are responsible for its diverse biological activities(28). The apalbumin 1 protein component isolated from manuka honey stimulates the production of proinflammatory cytokines, such as TNF-α and IL-6, through the toll-like receptor 4 signaling pathway(31).

Whereas, CH contains phenolic and alkaloids compounds, which are secondary metabolites derived from chestnut trees (32). Kynurenic acid (KYNA), which is a specific component of chestnut honey, was chosen as an indicator substance and analyzed using UPLC (Ultra -Performance Liquid Chromatography). As a result, KYNA was relatively abundant at 594.30 mg/kg in CH. KYNA has been studied for its diverse biological activities and has been reported to possess neuroactive activity(33). However, until recently, there had been no report on the innate immune-enhancing effect and antiviral efficacy of KYNA. Therefore, the antiviral effect of KYNA was investigated. As a result, it was found that IAV infection was inhibited when Raw264.7 macrophages were treated with a various concentration of KYNA (**Figure S3**). We propose that KYNA, a specific component of CH, inhibits influenza virus infection through the enhancement of innate immunity a potentially.

Our study revealed that CH-induced IFN production was mediated by the activation of specific signaling pathways. In particular, CH treatment led to the increased nuclear translocation of transcription factors IRF3/7 and NF-κB, which are the key regulators of antiviral IFN and proinflammatory cytokine production. This translocation was achieved through the activation of RIG-1 and MAVS signaling pathways.

Previous studies have reported increased frequency of influenza virus infection in RIG-1- and MAVS-deficient mice compared with that in WT mice, with high airway and lung viral titers. RIG-1 and MAVS pathways are susceptible to influenza viruses and mediate antiviral responses(11). MAVS localizes around the mitochondrial membrane and is activated by reactive oxygen species and calcium; moreover, it plays an essential role in the innate and acquired immunity through the inflammasome-related inflammatory responses and signal transduction processes. However, viral proteins can inhibit the activity of MAVS. In the H5N1 strain of IAV, the PB1-F2 protein suppresses the MAVS-mediated antiviral innate immunity by reducing the mitochondrial membrane potential, thereby inhibiting MAVS signaling(34). Although it is clear that cells and viruses employ multiple mechanisms to regulate MAVS post-transcriptionally and -translationally, a clearer understanding of MAVS activity in antiviral immunity may lead to the development of new drugs. When MAVS was knocked down using siRNA, influenza virus infection was induced, and viral infection was not inhibited by CH treatment. This suggests that CH inhibits viral infection through the MAVS signaling pathway. However, studies regarding the relationship between MAVS and mitochondria are insufficient. Further investigation of the mitochondrial function and role of MAVS is required.

In summary, our findings suggest that the antiviral effects of CH are mediated through enhanced innate immunity. CH stimulates the production of immune signaling molecules, including IFNs and proinflammatory cytokines, by activating RIG-1 and MAVS signaling pathways. These results provide insights into the molecular mechanisms underlying the antiviral properties of CH and its potential use as an immune-enhancing natural remedy against viral infections.

## Conclusions

CH protects cells from IAV infection by increasing the production and secretion of type-1 IFNs and proinflammatory factors, which stimulate an antiviral response involving the MAVS-mediated IFITM3 pathway. Taken together, CH prevents infection by upregulating the host immune response through a nonspecific immunologic mechanism. Thus, CH is a potential treatment for IAV infection and may be a useful immunomodulator.

## Data availability statement

The original contributions presented in the study are included in the article/**Supplementary Material**, further inquiries can be directed to the corresponding author.

## Ethics statement

The animal studies were approved by Laboratory Animal Center of Daegu-Gyeongbuk Medical Innovation Foundation (DGMIF- 22030103-01) and animal experiments (reference number #D-21–069) followed the guidelines of and were approved by the Animal Care and Use Committ ee of the Korea Institute of Oriental Medicine (KIOM, Daejeon, Korea). The studies were conducted in accordance with the local legislation and institutional requirements. Written informed consent was obtained from the owners for the participation of their animals in this study.

## Author contributions

E-BK and S-GK conducted the conceptualization, writing—original draft, methodology and validation. YK, BK and SH conducted the writing—original draft, methodology and validation. J-GC conceived the idea and designed the experiments, writing—original draft, methodology and validation. All authors read and approved the final manuscript.
