# Supplementary material for: Castanea crenata honey reduces influenza infection by activating the innate immune response
Source: Front Immunol. 2023 Aug 22;14:1157506. doi: 10.3389/fimmu.2023.1157506 (PMC10497975; doi:10.3389/fimmu.2023.1157506)
Supplement: Supplementary file 1 [file DataSheet_1.docx]

Supplementary Material

*Castanea crenata* honey reduces influenza infection by activating the innate immune response

**Eun-Bin Kwon^1^†, Se-Gun Kim^2^†, Young Soo Kim^1^, Buyun Kim^1^, Sang Mi Han^2^, Hye Jin Lee^2^, Hong Min Choi and Jang-Gi Choi^1^***

*** Correspondence:** Jang-Gi Choi, jang-gichoi@kiom.re.kr

#
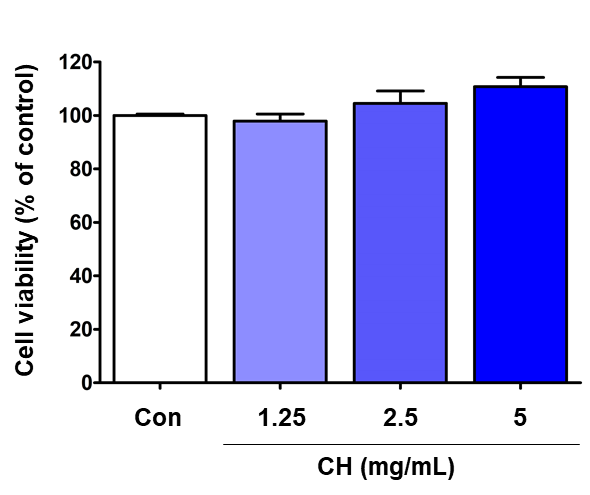


**Figure S1.** Effect of CH on cell viability. The cells were treated with various concentrations of CH for 24 h. Cell viability was assessed using an MTT assay. The experiments were performed three times independently.


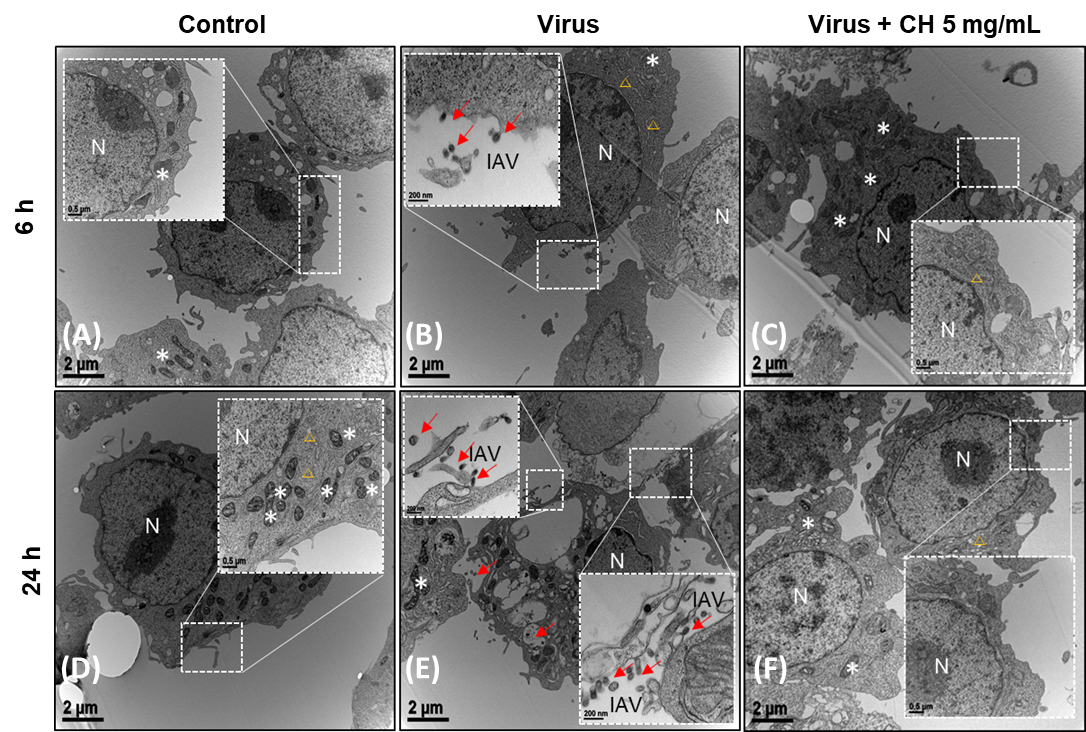


**Figure S2.** TEM images of IAV-infected Raw264.7 cells. The cells were pretreated with CH at 5 mg/mL for 24 h. After incubation, cells were harvested 6 and 24 h after IAV infection. (A) The control group at 6 h. (B) The virus-infected group at 6 h. (C) The cells were pretreated with CH at 5 mg/mL for 24 h and then infected with IAV for 6 h. (D) The control group at 24 h. (E) The virus-infected group at 24 h. (F) The cells were pretreated with CH at 5 mg/mL for 24 h and then infected with IAV for 6 h. Red arrow, IAV; white asterisk, mitochondria; N, nucleus; yellow triangle, ER.


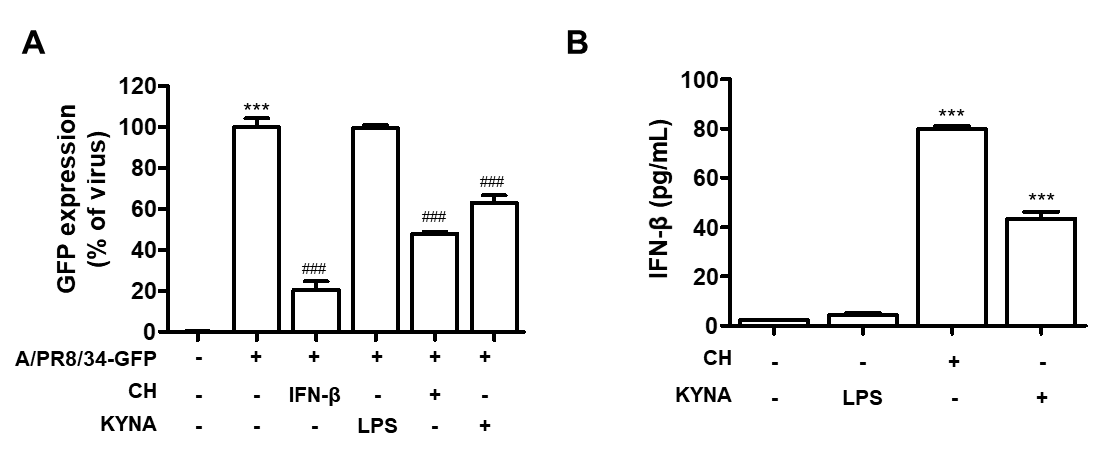


**Figure S3.** Comparison of antiviral effects on major compound and content of LPS in CH. (A) The cells were pretreated with 0.2 ng/mL LPS, 5 mg/mL CH and 100 μM KYNA or 1,000 units of IFN-β as a positive control for 24 h before infection with IAV-GFP. Detection of GFP using flow cytometry. (B) The cells were treated with 0.2 ng/mL LPS, 5 mg/mL CH and 100 μM KYNA for 24h. The supernatants were collected and centrifuged at 1500 rpm for 5 min at 4°C. The secretion of IFN-β were measured by ELISA kit. Bar graph (mean ± SEM) statistics were determined using one-way ANOVA with Tukey’s post hoc test. ^***^P < 0.001 compared with the untreated group (Con). ^###^P < 0.001 compared with the virus-infected group (Veh). CH, Chestnut honey; A/PR8/34-GFP, GFP encoding influenza A virus (A/PR8/34); LPS, lipopolysaccharides; IFN-β, Interferon beta; KYNA, Kynurenic acid.


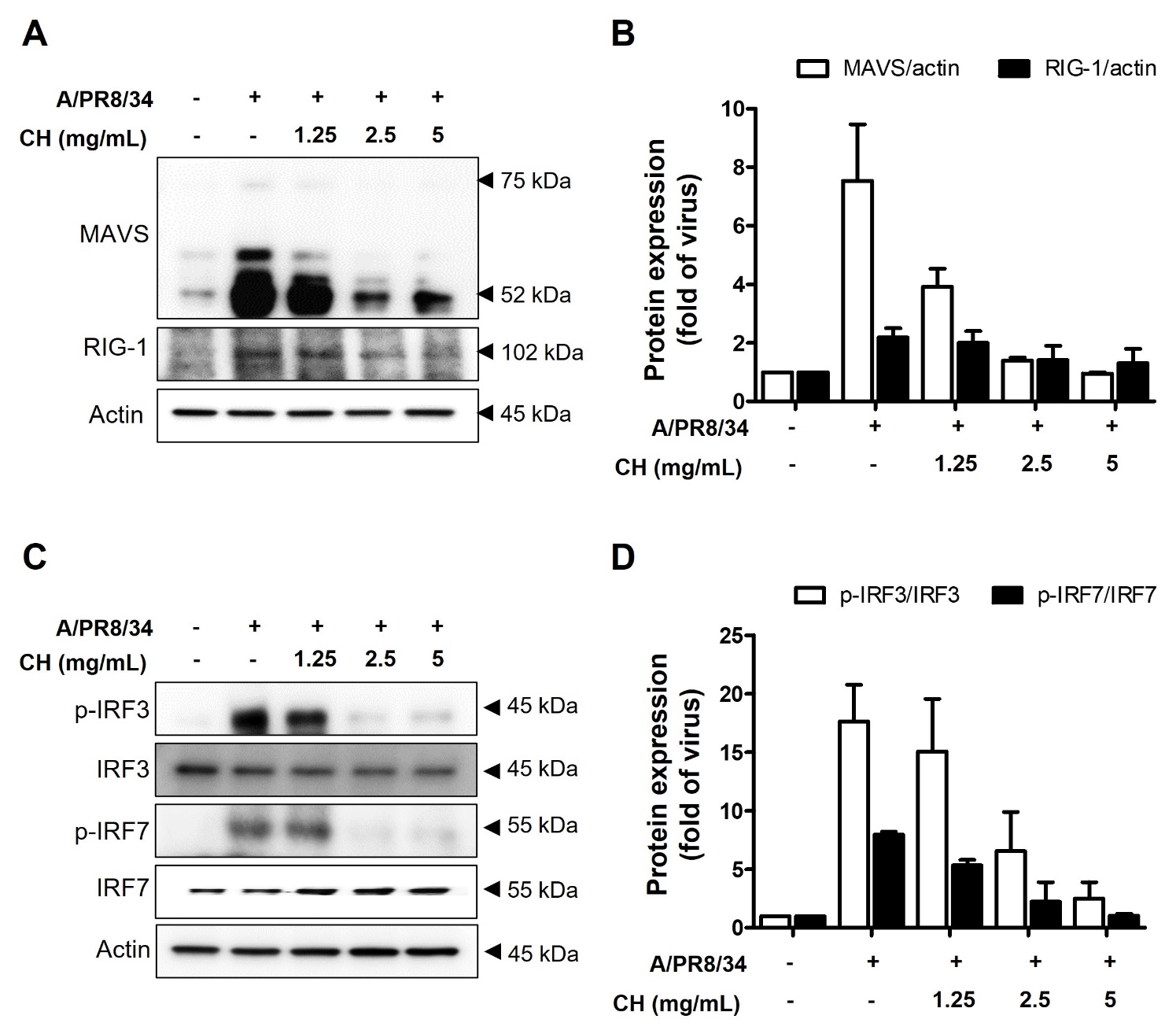


**Figure S4.** Effect of CH on virus induced inflammation. The cells were pretreated with CH for 24 h. After incubation, cells were harvested 24 h after IAV infection. The supernatants were collected and centrifuged at 1500 rpm for 5 min at 4°C. (A) Western blot analysis for expression of MAVS and RIG-1 using whole-cell lysates and (B) quantitative analysis of protein bands using the ImageJ software. (C) Expression of protein including phosphorylation IRF3 and IRF7 were analyzed by western blot. (D) Quantitative analysis of protein bands using the ImageJ software Bar graph (mean ± SEM) statistics were determined using one-way ANOVA with Tukey’s post hoc test. ^***^P < 0.001 compared with the untreated group (Con). ^###^P < 0.001 compared with the virus-infected group (Veh). CH: Chestnut honey, IAV: Influenza A virus, IFN: interferon


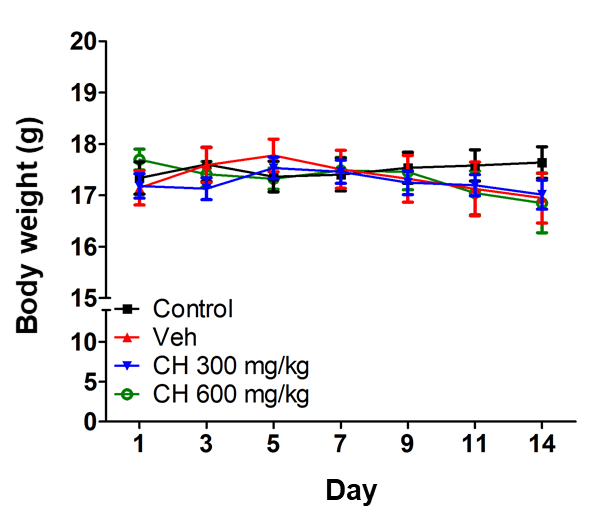


**Figure S5.** Weight during administration of CH for2 weeks. The 300 and 600 mg/kg CH was orally administered to mice for 2 weeks. Body weight were monitored daily until 14 days.

**Table S1.** Quantification of endotoxin content in honey sample

| Honey sample | LPS level  (EU/g of honey)* | | LPS level  (ng/g of honey)* | | LPS in diluted honey solutions  (ng/mL)** | |
| --- | --- | --- | --- | --- | --- | --- |
| chestnut honey | | 407 ± 25.17 | | 40.7 ± 2.52 | | 0.2 ± 0.01 |
| Oilseed rape honey | | 213 ± 65.06 | | 21.3 ± 6.51 | | 0.1 ± 0.03 |
| Acacia honey | | 263 ± 15.28 | | 26.3 ± 1.53 | | 0.12 ± 0.01 |

*LPS in undiluted honey, and **Honey diluted to 0.5% (w/v). Based on 10 EU = 1 ng endotoxin.
